# Light-induced irreversible structural phase transition in trilayer graphene

**DOI:** 10.1038/s41377-020-00412-6

**Published:** 2020-10-13

**Authors:** Jianyu Zhang, Jinsen Han, Gang Peng, Xi Yang, Xiaoming Yuan, Yongjun Li, Jianing Chen, Wei Xu, Ken Liu, Zhihong Zhu, Weiqi Cao, Zheng Han, Jiayu Dai, Mengjian Zhu, Shiqiao Qin, Kostya S. Novoselov

**Affiliations:** 1 Department of Physics, National University of Defense Technology, 410073 Changsha, China; 2 College of Advanced Interdisciplinary Studies, National University of Defense Technology, 410073 Changsha, China; 3 Hunan Key Laboratory of Super Micro-structure and Ultrafast Process, School of Physics and Electronics, Central South University, 410083 Changsha, China; 4 Quantum Design China (Beijing) Co., Ltd, 100015 Beijing, China; 5 Beijing National Laboratory for Condensed Matter Physics, Institute of Physics, Chinese Academy of Sciences, 100190 Beijing, China; 6 School of Physical Sciences, University of Chinese Academy of Sciences, 100049 Beijing, China; 7 Songshan Lake Materials Laboratory, Dongguan, 523808 Guangdong China; 8 Chongqing 2D Materials Institute, Liangjiang New Area, 400714 Chongqing, China; 9 Shenyang National Laboratory for Materials Science, Institute of Metal Research, Chinese Academy of Sciences, 110016 Shenyang, China; 10 School of Material Science and Engineering, University of Science and Technology of China, 230026 Anhui, China; 11 State Key Laboratory of Quantum Optics and Quantum Optics Devices, Institute of Opto-Electronics, Shanxi University, 030006 Taiyuan, China; 12 Department of Materials Science and Engineering, National University of Singapore, Singapore, 117575 Singapore

**Keywords:** Optical properties and devices, Raman spectroscopy

## Abstract

A crystal structure has a profound influence on the physical properties of the corresponding material. By synthesizing crystals with particular symmetries, one can strongly tune their properties, even for the same chemical configuration (compare graphite and diamond, for instance). Even more interesting opportunities arise when the structural phases of crystals can be changed dynamically through external stimulations. Such abilities, though rare, lead to a number of exciting phenomena, such as phase-change memory effects. In the case of trilayer graphene, there are two common stacking configurations (ABA and ABC) that have distinct electronic band structures and exhibit very different behaviors. Domain walls exist in the trilayer graphene with both stacking orders, showing fascinating new physics such as the quantum valley Hall effect. Extensive efforts have been dedicated to the phase engineering of trilayer graphene. However, the manipulation of domain walls to achieve precise control of local structures and properties remains a considerable challenge. Here, we experimentally demonstrate that we can switch from one structural phase to another by laser irradiation, creating domains of different shapes in trilayer graphene. The ability to control the position and orientation of the domain walls leads to fine control of the local structural phases and properties of graphene, offering a simple but effective approach to create artificial two-dimensional materials with designed atomic structures and electronic and optical properties.

## Laser on trilayer graphene: stack switch with a difference

Heat from a laser changes how atoms stack up within trilayer graphene, providing a simple and effective approach for fabricating new materials with interesting optical and electronic properties. Mengjian Zhu of China’s National University of Defense Technology and colleagues used laser light to switch regions within trilayer graphene from one type of atomic layering to another. This is interesting, as the properties of trilayer graphene vary depending on how its atoms are stacked up. Zhu and his colleagues found that heat from the laser manipulated the dividing walls separating differently stacked atomic regions within the graphene flakes. This led to a gradual switch from one type of atomic stacking to another, changing the graphene’s properties. The finding could lead to applications for optical storage media and photonic devices.

## Introduction

The stacking configuration of layered materials plays an important role in determining their electronic and optical properties. Fascinating phenomena, such as Hofstadter’s butterfly, Mott insulators, ferromagnetism, and unconventional superconductivity, can also emerge in van der Waals heterostructures by carefully controlling the layer stacking sequence^1–8^. In the case of trilayer graphene (TLG), there are two common stacking configurations: the top layer may lie directly above the bottom layer (denoted as Bernal or ABA stacking) or may instead lie above the center of the hexagon of the bottom layer (denoted as rhombohedral or ABC stacking)^9,10^. Due to different interlayer electron interactions and distinct crystal symmetry, it has been shown that ABA-stacked TLG and ABC-stacked TLG exhibit significantly different physical properties. From the perspective of the electronic band structure, ABA-stacked TLG is a semimetal with a gate-tunable band overlap between the valence and conduction bands, whereas the ABC-stacked TLG is a semiconductor with an electrically tunable band gap^11–15^. In TLG flakes containing both ABA and ABC stacking, there are domain walls between the phases, consisting of a localized strain soliton in which the carbon atoms of one graphene layer shift by the carbon–carbon bond distance^16,17^. Such domain walls in TLGs have attracted much interest because of their intriguing physical properties. For example, optically, soliton-dependent reflection of graphene plasmons at the domain walls has been experimentally observed^18^. Electrically, the domain walls are predicted to host topological edge states and ballistic transport and can also produce in-plane metal–semiconductor (ABA–ABC) homojunctions in TLG^19–21^.

Previous reports have shown that applying molecular absorption or an external electric field can drive the stacking order transition and generate domain wall motion in graphene layers^17,22,23^. There is inevitable residue on graphene with molecular doping, which hinders the properties of graphene. Applying an electrical field or strain usually leads to global control of the stacking order phase and hinders precise manipulation of the local structure. An alternative way to change the stacking configuration is by applying a local mechanical force. For example, a previous study demonstrated that domain walls in TLGs can be moved by mechanical stress exerted through an atomic force microscopy (AFM) tip^16^. The domain walls are invisible in conventional AFM topography, and studies must rely on near-field infrared nanoscopy measurements. However, a simple and controllable approach to engineer the stacking phase and domain walls into designed atomic structures is still lacking.

Here, we experimentally demonstrate that the stacking order in TLG can be switched from ABC to ABA by local heating enabled through laser irradiation. The light-induced stacking phase transition in TLG is directly visualized using Raman mapping and near-field nanoscopy imaging. By controlling the movement of the laser beam with considerable flexibility and precision, we are able to reshape the domains and manipulate the position and orientation of the domain walls in the TLG. We attribute the laser-induced local heating effect as the main driving force of the ABC-to-ABA phase transition. The activation energy is determined by Raman spectroscopy measurements and thermal annealing experiments and is consistent with the calculated energy barrier height of approximately 40 meV determined by density functional theory (DFT) calculations. The electronic and optical properties of TLG strongly depend on the stacking configuration. Therefore, the ability to achieve fine control of the local stacking configuration and manipulate the domain walls by a simple and clean approach opens the way to new devices with fascinating functionalities, such as multilevel optical switch and phase-change memory.

## Results and discussion

**Fig. 1:**
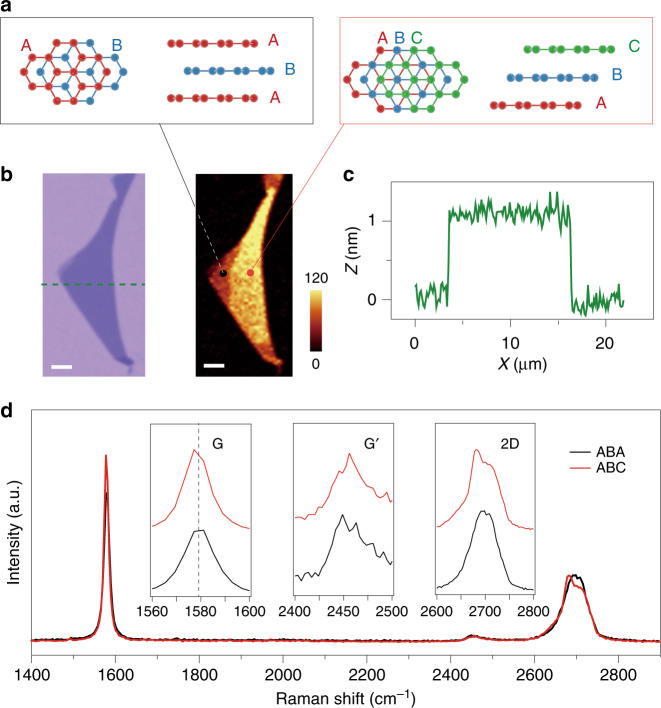
Characterizations of mechanically exfoliated TLG. **a** Schematics of graphene trilayers with ABA stacking (left) and ABC stacking (right) configurations. The bottom, middle, and top layers are labeled with different colors. **b** Optical microscopy image of TLG sample #2 and the corresponding Raman mapping of the integrated G band intensity. The darker domain indicates the ABA-stacked TLG, and the brighter domain was defined as ABC stacking. The scale bars are 4 μm, and the color bar shows the integrated Raman intensity. **c** AFM height profile of TLG measured along the green dashed line in the optical image in (**b**). **d** Raman spectra of TLG taken from different regions marked in the Raman mapping image in (**b**). The insets show magnified spectra of TLG: G band (1560–1600 cm^−1^), G^’^ band (2400–2500 cm^−1^), and 2D band (2600–2800 cm^−1^)

The schematics in Fig. 1a shows the crystalline structures of TLG with ABA and ABC stacking orders. The atoms of the topmost layer in ABA-stacked TLG lie exactly above of those of the bottom layer, whereas in ABC-stacked TLG, the sublattice of the top layer lies above the center of the hexagons in the bottom layer. There is a parallel shift of exactly one carbon honeycomb between the topmost layers in the two allotropes^9^. Graphene trilayer was obtained by mechanical exfoliation and confirmed by optical contrast, and the thickness (~1.2 nm) was determined by AFM, as shown in Fig. 1b, c. Raman spectroscopy has been demonstrated to be an accurate and effective method to distinguish the ABA and ABC stacking structures in TLG^10,24–26^. TLG flakes show very uniform optical contrast without any visible domain walls or wrinkles. However, the Raman mapping exhibits two distinct domains with significant contrast due to the different stacking orders in TLG, as shown in Fig. 1b (here, integrated G band intensity is presented)^10,25^. The darker domain was identified as ABA-stacked TLG, and the brighter domain was identified as ABC stacking. Raman spectra of the two different domains are plotted for comparison in Fig. 1d. The spectra are different from one another in at least three ways: first, the 2D band of ABC-stacked TLG shows more asymmetric features with an enhanced peak and shoulder compared with the symmetric feature shown in ABA-stacked TLG; second, the G band of the ABC-stacked domain is redshifted by ~1 cm^−1^ compared with that of the ABA-stacked domain; and third, the G’ band of the ABC-stacked TLG domain also exhibits more asymmetric features than its ABA-stacked counterpart^10,25,27^.

We will focus on the integrated G band intensity mappings (Fig. 1b), which show the lowest noise level (mappings of bandwidth of G bands and 2D yield consistent shape of domains, as shown in Fig. S1). In this work, we prepared 211 TLG flakes. Among them, 147 flakes are pure ABA-stacked TLG, and the remaining 64 flakes have coexisting ABA- and ABC-stacked domains, as shown in Fig. S2. The proportion of ABC stacking in TLG is ~15%, consistent with previous reports^10,28^.

**Fig. 2:**
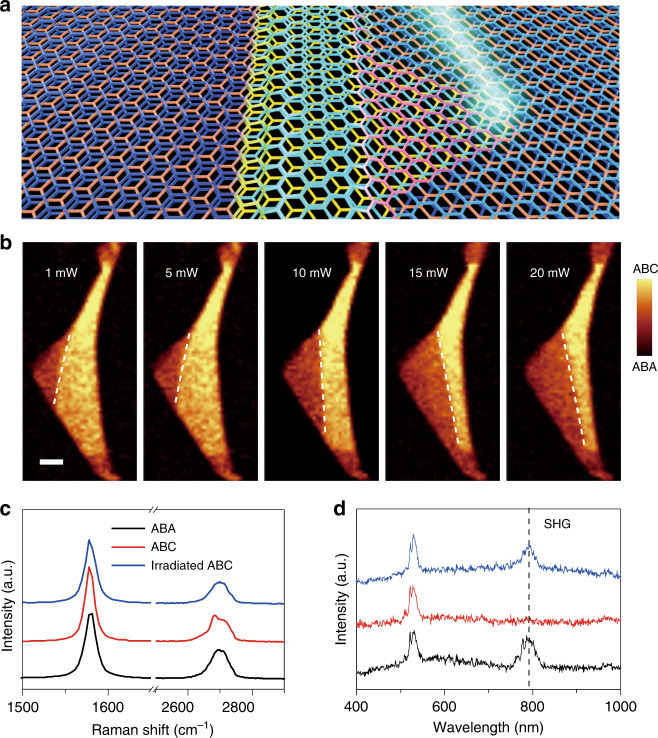
Light-induced ABC-to-ABA structural phase transition in TLG. **a** Artistic view of the laser-driven stacking order transformation in TLG. The ABA-stacked domain (left) and ABC-stacked domain (right) are separated by a domain wall (middle). **b** Raman mappings of the integrated G band intensity of TLG sample #2 after laser irradiation at various laser powers from 1 to 20 mW. The exposure time was 12 min for each laser scan. The white dashed lines indicate the gradual movement of the ABA/ABC domain wall under laser irradiation. The laser scan direction is from left to right and then from top to bottom. The scale bar is 4 μm. **c** Raman spectra and optical SHG responses (**d**) of the ABA-stacked domain, ABC-stacked domain and laser-irradiated ABC-stacked domain with a power of 20 mW. The dashed vertical line in (**d**) marks the SHG response of TLG at ~790 nm

An attractive target for optical materials is to find a system that shows the structural phase transition triggered by external stimulation of light^29–31^. Laser irradiation has been demonstrated as an effective method to induce structural phase transitions in two-dimensional materials, for instance, laser-driven 2H-to-1T’ phase transitions in few-layer MoTe_2_^32–34^. Here, we extend this methodology to control the stacking order transformation in TLG. A continuous laser beam was scanned over the TLG sample under ambient conditions, as schematically shown in Fig. 2a (see the “Materials and methods” section for more details). The sample was illuminated by a laser with different powers from 1 to 20 mW. First, the laser beam moved from left to right to finish one line scan. After that, the laser returned to the left and moved downward to start the next line scan until all scans are completed. After finishing each laser scan, the sample was again characterized by Raman mapping with a laser power of 1 mW. Figure 2b summarizes the Raman mappings of the integrated G band intensity, showing the lapsed dynamic process of the phase transition from ABC stacking to ABA stacking. The domain wall started to move from the ABA-stacked domain to the ABC-stacked domain under 10 mW laser irradiation. As the laser power increased, the domain wall gradually shifted from left to right, showing a reduced ABC stacking area and an expanded ABA stacking region. The Raman mappings of the integrated 2D band intensity show the same transformation process, as shown in Fig. S3. Furthermore, we found that if the laser scan zone contained the domain walls, then the ABC-to-ABA stacking order transition always initiated from the domain wall rather than randomly occurring in the TLG. However, the ABC-to-ABA phase transition can also occur in the pure ABC-stacked TLG region, as shown in Fig. S4. Notably, the light-induced ABC-to-ABA phase transition was highly reproducible in many other TLG samples, regardless of the geometric shapes of the domain walls and the angle between the laser scanning direction and the domain walls, as shown in Fig. S5. We noticed that the movement of the ABA/ABC domain walls in TLG was similar to the bilayer graphene case. In bilayer graphene, the AB/AC stacking boundaries were observed as nanometer-wide strained channels, mostly in the form of ripples, producing smooth low-energy transitions between the two different stacks^35^.

Figure 2c shows the significant changes in the Raman spectra of TLG before and after laser irradiation. It was evident that the 2D band of the ABC domain became more symmetric after laser irradiation, which agrees with the 2D band features of the initial ABA-stacking domain. To further confirm the nature of the ABC-to-ABA structural transition, we carried out optical SHG measurements (see “Materials and methods”), which have been shown to be a reliable characterization method for crystal structures of two-dimensional materials lacking inversion symmetry, thus being very sensitive to the stacking sequence. A previous study demonstrated a strong SHG response in ABA-stacked non-centrosymmetric TLG, while this response vanished in ABC-stacked TLG, which preserves the inversion symmetry^36^. We observed a similar SHG response for the initial ABA- and ABC-stacked domains, as shown in Fig. 2d. The SHG peak appears in the spectrum of the area where the ABC-stacked domain is located after laser irradiation, suggesting light-induced disruption of the inversion symmetry due to the ABC-to-ABA-stacking order transformation in the TLG.

**Fig. 3:**
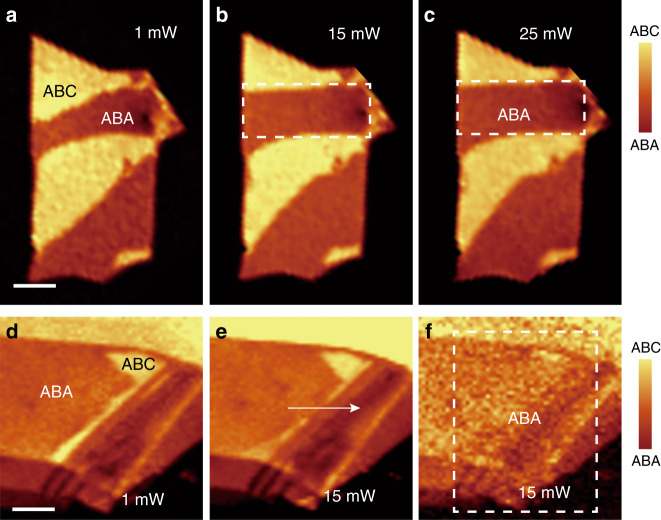
Versatile manipulation of domain walls in TLG. **a–c** Reshaping the ABA/ABC domain walls in TLG. Raman mappings of the integrated G band intensity of TLG sample #63 under laser irradiation with different powers from 1 to 25 mW. The exposure time is 11 min for each laser scan. **d–f** Creation and erasure of ABA/ABC domain walls in TLG. Raman mappings of the integrated G band intensity of sample #14. The exposure times are 6 min in **e** and 11 min in **f**. The white dashed rectangles represent the area scan of the laser, and the arrow indicates the line scan of the laser. The scan direction of the laser is from left to right and then from top to bottom. The scale bars are 4 μm

Laser irradiation further enables phase patterning in TLG by local control over the geometries of the ABA- and ABC-stacked domains. Versatile manipulation of the domain walls is accomplished by our technique, including reshaping and erasure of the domain walls, as well as creation of closed-loop domain walls, as shown in Fig. 3. The laser-irradiated ABC-stacked domain was found to transform to an ABA-stacked domain, while the nonirradiated region retains the initial ABC stacking phase without change. By area scanning over the desired region, the shape of the domain wall is redefined by laser irradiation (Fig. 3a–c). A similar execution area scan of the laser is employed to erase the domain walls in the TLG (Fig. 3d, f). We can also create closed-loop domain walls with an ABC-stacked domain inside by cutting through an existing domain (Fig. 3d, e). Based on this technique, one can create new domains with arbitrary shapes and can manipulate the position and orientation of the domain walls. Such ability to control the geometry of domain walls in a desired area with a submicron resolution (determined by the diameter of the laser spot) will lead to fine control over the structural phases and topological states in graphene and other two-dimensional quantum materials.

**Fig. 4:**
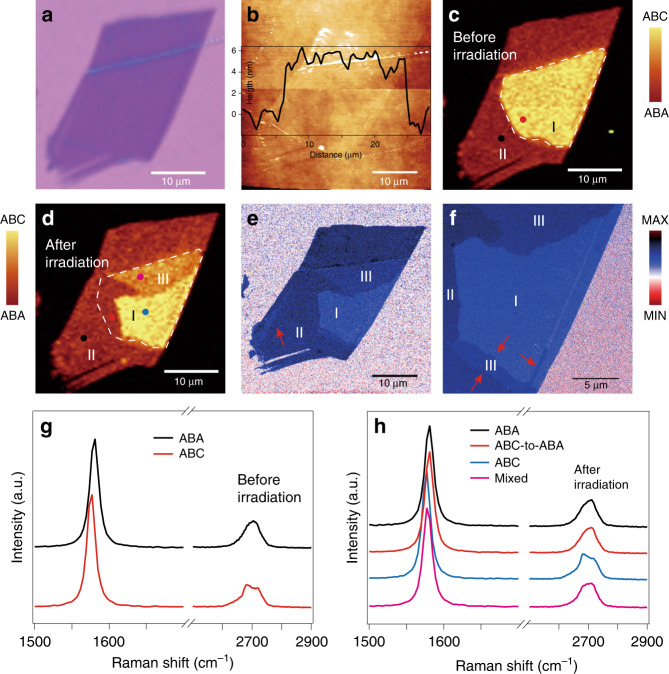
Raman mapping and s-SNOM imaging of the light-induced structural phase transition in MLG. **a** Optical microscopy image of MLG sample #125. **b** AFM image and height profile of graphene. **c** Raman maps of the integrated G peak intensity (position: 1576 cm^−1^, width: 5 cm^−1^) before laser irradiation and (**d**) after laser irradiation. The laser power is 20 mW, and the exposure time is 34 min. **e** s-SNOM image of graphene after laser irradiation. **f** Magnified s-SNOM image of graphene. Graphene domains with different stacking orders show different contrasts in the s-SNOM image. The marked regions I, II, and III correspond to ABC stacking, ABA stacking and mixed ABC + ABA stacking domains, respectively. The red arrows in (**e**, **f**) highlight the additional mixed ABC + ABA stacking domains that were not resolved in the Raman maps. **g** Raman spectra of different graphene regions taken from the marked solid dots before laser irradiation and (**h**) after laser irradiation

In addition to TLG, thicker multilayer graphene (MLG) also exhibits ABA and ABC stacking configurations. We exfoliated MLG flakes onto an oxidized silicon substrate and combined optical contrast measurements, AFM, and Raman spectroscopy to determine the number of layers. The optical microscopy image of MLG sample #125 is shown in Fig. 4a. Despite the uniform thickness (~2.5 nm, 6 ± 1 graphene layers) and featureless morphology (Fig. 4b), the Raman map of the integrated G peak intensity (laser power: 2 mW) exhibits two regions with strikingly different contrast, as shown in Fig. 4c. According to previous reports, these distinct regions are thought to also arise from the different stacking sequences in the MLG. We further probe the details of the Raman spectrum of each region in the MLG, as shown in Fig. 4g. The 2D peaks clearly show the line shape characteristics of ABA (black) and ABC (red) stacking. In addition, the G peak is ~4 cm^−1^ lower than that in the ABA-stacking domain, which is also a characteristic of ABC-stacked MLG.

After Raman mapping, the whole flake was scanned by a laser beam. After laser illumination, we again plotted the Raman map of the integrated G peak intensity, as shown in Fig. 4d. There are two significant changes in the irradiated MLG. First, the domain wall moved, and the area of the ABC domain shrank. Second, a new region of mixed ABA and ABC stacking formed after laser irradiation, marked by purple dots in Fig. 4d. To further understand the origin of these three regions, we analysed the Raman spectrum of each region in more detail, as shown in Fig. 4h. It is evident that a part of the ABC domain has been completely transformed into ABA stacking (red dot) and that another part of ABC-stacked graphene transformed into mixed ABA and ABC stacking (purple dot).

Although we performed the Raman measurements with great care, the resolution is <1 μm but still larger than 500 nm due to the limit of the laser spot size (~0.6 μm). To achieve better resolution of the domain walls in graphene, we employed scattering-type scanning near-field optical microscopy (s-SNOM) to directly image the stacking structure and domain walls in the graphene samples (see “Materials and methods”). In trilayer or MLG, ABA- and ABC-stacked domains give different infrared responses due to their different electronic band structures, resulting in different contrast in the s-SNOM image, as shown in Fig. 4e. Domain walls are observed in the transitional regions between different stacking domains. The s-SNOM image of irradiated MLG shows features of the domain walls that are highly consistent with the Raman maps but exhibits a higher resolution of approximately tens of nanometers. Additional mixed ABC + ABA stacking domains and domain walls are clearly resolved in the detailed s-SNOM image (Fig. 4f), which was not observed in the Raman measurements. The s-SNOM imaging of domain walls after additional laser irradiation is shown in Fig. S6.

To understand the origin of the laser-induced ABC-to-ABA phase transition in graphene, we summarized the positions of the G peaks (*ω*_G_) and 2D peaks (*ω*_2D_) of TLG, as shown in Fig. S7. Both *ω*_G_ and *ω*_2D_ undergo downshifts under laser irradiation with power ranging from 1 to 50 mW. To analyse the effect of laser-induced local heating and strain, we plotted *ω*_G_ vs. *ω*_2D_. In contrast to the reported upshift of *ω*_G_ and *ω*_2D_ due to strain relaxation, both *ω*_G_ and *ω*_2D_ downshift under laser irradiation in our experiments^37,38^. This result implies that laser-induced local heating is essential for the phase transition in TLG, and thus, the stacking order switch is thermal. To further exclude the effect of local strain, we performed laser irradiation experiments in TLG on Al_2_O_3_. The thermal expansion coefficient of Al_2_O_3_ is ~5 × 10^−6^ K^−1^, an order of magnitude higher than that of SiO_2_, which may lead to different local strains in laser-irradiated graphene. However, our results show that the light-induced ABC-to-ABA structural phase transition also occurs in TLG on the Al_2_O_3_ substrate, as shown in Fig. S8. In addition, we observed consistent light-induced stacking order transitions in graphene with different exposure times (Fig. S9) and different laser wavelengths (Fig. S10).

**Fig. 5:**
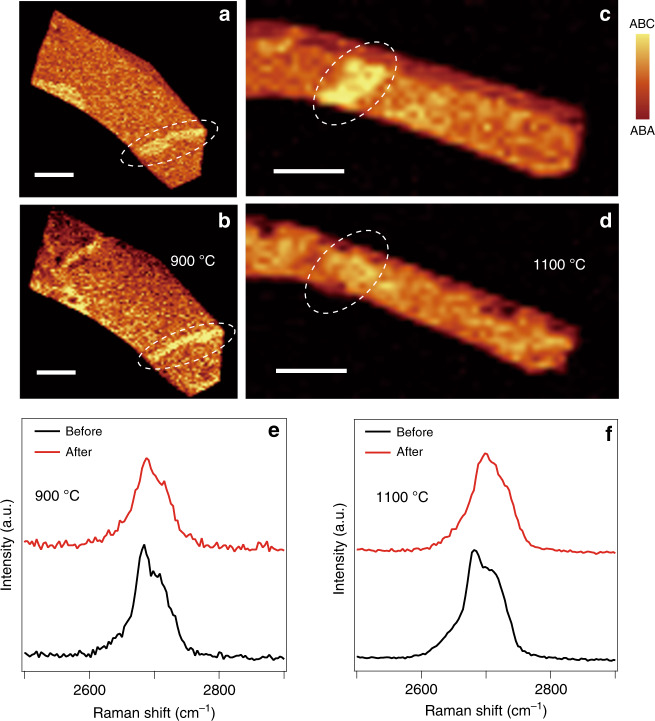
Thermal annealing-induced ABC-to-ABA structural phase transition in TLG. **a**, **b** Raman mappings of the integrated G band intensity of sample #98 before (**a**) and after (**b**) annealing at 900 °C for 8 h. The scale bars are 6 μm. **c**, **d** Raman mappings of the integrated G band intensity of sample #96 before (**c**) and after (**d**) annealing at 1100 °C for 8 h. The scale bars are 9 μm. The white dashed zones highlight the ABC-stacked domains in (**a–c**), which disappear in (**d**) after annealing at 1100 °C. **e**, **f** Raman spectra showing 2D bands of ABC-stacked domains before annealing and after annealing at 900 °C and 1100 °C, respectively

We now consider the laser-induced local heating effect to be the main driving force of the structural phase transition in TLG. To verify the role of thermal activation, we performed annealing of TLG in an argon atmosphere (see “Materials and methods”). In fact, Laves and Baskin were able to produce ABC graphite initiated by unidirectional pressure associated with shear force half a century ago^39^. They also observed transformation from ABC to ABA stacking in graphite by heating the samples at 1300 °C for 4 h. However, the stacking order transformation has not yet been observed in TLG by thermal heating up to 800 °C^10^. Here, we demonstrated the annealing-induced ABC-to-ABA structural phase transition in TLG at 1100 °C for 8 h. As shown in Fig. 5a, b, the Raman mappings do not show any significant change after annealing at 900 °C for 8 h, whereas the ABC-stacked domain completely transforms to the ABA-stacked domain after annealing at 1100 °C, as shown in Fig. 5c, d. The comparison between the Raman spectra and 2D bands before and after thermal annealing further confirms the ABC-to-ABA phase transition at 1100 °C, as shown in Fig. 5e, f.

**Fig. 6:**
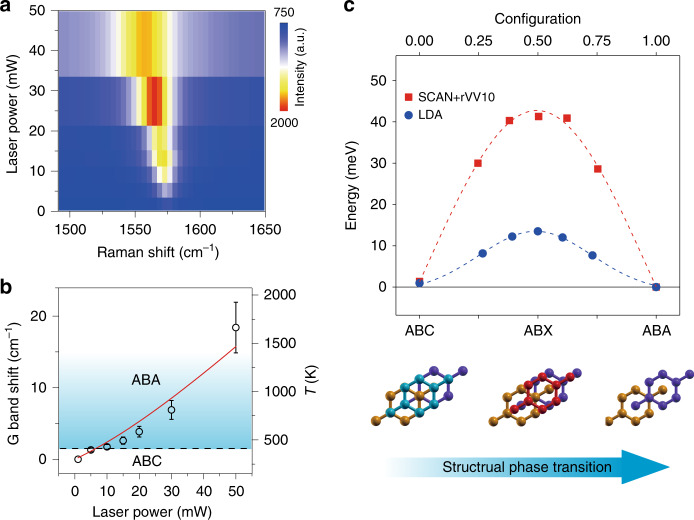
Determining the energy barrier of the structural phase transition in TLG. **a** 2D color plot of Raman shifts of G bands as a function of applied laser power. **b** The change in Raman shifts of the G bands (left axis) and the determined lattice temperature (right axis) of TLG. The data are from sample #2. The error bars are determined by the uncertainty of the coefficient *γ* (0.011 cm^−1^ K^−1^ ~ 0.016 cm^−1^ K^−1^). The red solid curve is the lattice temperature of TLG calculated by the heat diffusion equation. The horizontal black dashed line marks the threshold temperature *T*_ts_ ~ 430 K for the structural transition in TLG under 10 mW laser irradiation. **c** DFT calculations of the minimum energy transition path of TLG from ABC stacking to ABA stacking using the NEB method. The red squares represent the calculation performed by means of the SCAN + rVV10 functional, while the blue dots represent the calculation performed by means of the LDA functional. The dashed curves are visual guides obtained by Gaussian fitting. The schematics at the bottom illustrate three different stacking configurations of TLG and the process of ABC-to-ABA structural phase transition

During laser illumination, light absorption significantly increases the lattice temperature of TLG, which was measured in situ by Raman G band shifts. Figure 6a plots a color map of the G band position as a function of laser power, showing a continuous redshift with increasing laser power. We estimated the steady-state temperature *T* of TLG under laser irradiation using an established coefficient *γ* (0.011 cm^−1^ K^−1^ < *γ* < 0.016 cm^−1^ K^−1^) between the G band shift (Δ*v*) and lattice temperature of TLG: *T* = 300 + Δ*ω*_*G*_/*γ*, where Δ*ω*_*G*_ is the laser heating-induced downshift of the Raman G peak^40–43^. As shown in Fig. 6b, *T* monotonically increases as a function of laser power, reaching ~600 K under 20 mW laser irradiation. According to the Raman mappings in Fig. 2b, the phase transition initiates at 10 mW, and the corresponding temperature is approximately 430 K (see Fig. S11). We defined this temperature as the threshold temperature *T*_ts_ of the ABC-to-ABA phase transition in TLG. Interestingly, the deduced value of *T*_ts_ ~ 430 K in this work is consistent with a previous report that the stacking transition in rhombohedral graphite (7.5 nm thickness) starts to occur at ~500 K by Joule heating^44^. We notice that the *T*_ts_ value obtained from Raman spectroscopy is lower than that determined from the annealing experiments. We attribute this discrepancy to the defects induced by high-temperature annealing, which might pin the ABC stacking phase and raise the energy barrier for the phase transition^45,46^.

We can simulate the lattice temperature of TLG under laser irradiation using the three-dimensional finite element method to solve the heat diffusion equation (see “Materials and methods”). The calculated lattice temperature is in good agreement with that determined from Raman spectroscopy, as shown in Fig. 6b. Notably, we did not observe any Raman signature of defects in TLG after laser irradiation below 20 mW. As shown in Fig. S12, the D band only appears at the edges of TLG under 50 mW laser irradiation^47,48^. In addition, the Raman spectrum of irradiated graphene still shows the characteristics of TLG without any signature of bilayer or monolayer graphene. We attribute the absence of laser-induced defects and thinning effects in graphene to the relatively low power density of the laser and the presence of the SiO_2_/Si substrate. The substrate plays a crucial role as a heat sink for graphene, providing additional channels for heat dissipation.

To gain a further understanding of the physical mechanism of the ABC-to-ABA structural phase transition in TLG, we performed climbing image nudged elastic band (CI-NEB) calculations based on DFT (see “Materials and methods”)^49^. Figure 6c shows the calculated minimum energy pathway of TLG from ABC stacking to ABA-stacking configurations. There are two energy minima, corresponding to the two stable phases of TLG: ABA- and ABC-stacked TLG. It is also evident that ABA-stacked TLG exhibits a lower energy (~2 meV per unit cell) than ABC-stacked TLG. This is consistent with previous reports that ABA stacking is more thermodynamically stable than ABC stacking^13,22^. There is a significant energy barrier during the ABC-to-ABA phase transition. It should be noted that in the process of CI-NEB calculations, we did not fix any position of the atom, and the search for the barrier is promised by the CI-NEB method^50^. It is interesting that in the final transition path, the configuration at the highest energy corresponds to a particular stacking structure: the topmost layer lattice is parallelly shifted by half a carbon–carbon bond length compared with the top layer of ABC-stacked TLG, as shown in the schematic of Fig. 6c bottom. We named this unstable structure ABX stacking.

The simulation demonstrates that there is a relative conservation of energy when shifting only the topmost layer in the transition from ABC to ABA stacking. However, the change in the structure during the whole transition path is complicated, as the interlayer distance and the structures of the total three layers can be different. The final calculated energy difference Δ*E* between ABX and ABC stacking is approximately 40 meV (~508 K), as determined by the SCAN + rVV10 functional, which considers the nonlocal interactions, including van der Waals forces, between different layers in the TLG. Considering the thermal activation energy as *E*_a_ = k_B_*T* (k_B_ the Boltzmann constant), the above values are quite consistent with the estimated threshold temperature *T*_ts_ ~ 430 K for the light-induced phase transition in TLG determined in this work and with the *T*_ts_ ~ 500 K for the Joule heating-induced stacking transition in rhombohedral graphite reported in a previous study^44^. The total energy of TLGs with different stacking was also calculated, as shown in Table S1 in the Supplementary Information. Based on the above analyses, we can attribute the physical mechanism of the light-induced phase transition in TLG to the thermally activated parallel slipping and rearrangement of carbon atoms of the topmost layer of TLG caused by the laser local heating effect.

## Conclusions

In summary, this work explores whether laser irradiation of TLG results in a structural phase transition from ABC stacking to ABA stacking. The stacking order transformation was confirmed by significant changes in the Raman spectra and nonlinear optical SHG response of TLG. The light-induced phase transition was found to be a gradual changing process and to always initiate at the ABA/ABC domain walls. Versatile manipulation of the domain walls was accomplished by our technique, including reshaping and erasure of the domain walls, as well as creation of closed-loop domain walls. We were also able to observe the ABC-to-ABA structural phase transition by thermal annealing, highlighting the laser heating effect as the major driving force of the stacking order transition in TLG. The DFT simulations considering the van der Waals interaction suggested an energy barrier of ~40 meV for the structural phase transition due to the energy difference between the initial ABC stacking structure and an intermediate state. Our results reveal the physical mechanism of the light-induced structural phase transition in TLGs, which sheds light on the realization of reversible stacking transitions, as well as polymorphism engineering of two-dimensional material devices with new functionalities, including optical storage media, optically configurable metasurfaces, and photonic devices.

## Materials and methods

### Sample preparation

Graphene flakes were mechanically exfoliated from graphite bulk crystals (flaggy graphite, purchased from NGS company, Germany) onto a substrate with a 290 nm SiO_2_ capping layer on top of heavily doped silicon. We verified the TLG structure by optical microscopy, Raman spectroscopy and AFM. After characterization, we irradiated the TLG with a continuous laser beam using the scan mapping functional of a Raman spectrometer (Witec Alpha 300R). The wavelength of the laser was 532 nm, and the laser power was adjusted from 0 to 50 mW. For the thermal annealing experiments, TLG samples were placed in a quartz tube of a furnace and heated from room temperature to 1100 °C at a heating rate of 4 °C/min. The samples were held at 1100 °C for 8 h and then cooled to room temperature at a rate of 5 °C/min. During annealing, the samples were protected by a pure argon atmosphere under a low pressure of 30 Pa.

### Characterization

Raman spectroscopy of TLG was performed by means of a commercial confocal Raman spectrometer (Witec Alpha 300R). The laser wavelength was 532 nm, and the diameter of the laser spot was ~0.6 μm. The spectral resolution of the Raman spectra was 1 cm^−1^ using a grating of 600 grooves per mm. We calibrated the Raman spectra by the 520 cm^−1^ Raman peak of the silicon substrate. To avoid any heating effect, the laser power was fixed at 1 mW. We measured the nonlinear optical response of the TLG sample using a home-built setup. A femtosecond laser was coupled to the sample by a single-mode fiber with a spot diameter of ~4 μm. The output power of the femtosecond laser was 10 mW, the center wavelength was 1.57 μm, and the pulse width of the laser was 130 fs. The light emission from TLG was collected by an objective lens and then focused to a single-mode fiber and finally coupled to an optical spectrum analyser (Yokogawa AQ6370). To achieve better resolution of the domain walls in graphene, we employed scattering-type scanning near-field microscopy (s-SNOM, neaspec GmbH) to directly image the stacking structure and domain walls in the graphene samples. s-SNOM enables imaging in the infrared regions at a spatial resolution of ~10 nm. The s-SNOM measurement was based on tapping-mode AFM. An infrared incident light beam (*λ* = 10.6 μm) was focused onto the apex of a conductive AFM tip (Arrow NCPt, nanoWorld). To collect the scattered light that carries local optical information of graphene samples, we used a cooled HgCdTe detector placed in the far field. During the measurements, we recorded the near-field images simultaneously with the topography information.

### Temperature simulation

To investigate the temperature of TLG under laser irradiation, we use the three-dimensional finite element method to solve the heat diffusion equation −∇·(*κ*∇T) = *q*, where *κ* is the thermal conductivity and $q \,=\, I\alpha \cdot {\mathrm{exp}}\left( { - \left( {2r^2} \right)/\left( {r_0^2} \right)} \right)$ is the heat inflow per unit area owing to laser excitation, where *I* is the laser intensity and *α* is the absorptance of TLG (6.9%). *r*_0_ is the radius of the laser spot (0.3 µm). In our simulation, the thermal conductivities of SiO_2_ and Si were 1.4 and 50 W/(m K), respectively^51^. The thermal conductivity of graphene was determined by the temperature of acoustic phonon *κ*(*T*_*ap*_) = *κ*(*T*_0_)(*T*_0_/*T*_*ap*_)^*γ*^, where *κ*(*T*_0_) = 450 W/(m K), *γ* = 1, with *T*_0_ = 300 K^52^. The interface between graphene and the underlying SiO_2_ was modeled with a thermal resistance of 2 × 10^−8^ m^2^ K/W^51^. A convective heat flux boundary condition was used in our model to describe the heat transfer between air and the upper boundary. The heat transfer coefficient was set as 5 W/m^2^ K. A fixed temperature (300 K) boundary condition was used at the boundary of the substrate. The temperature measured by Raman spectroscopy was a weighted average of the temperature inside the laser spot. In our simulation, we defined the average temperature as^53^:

$$T_{\mathrm{s}} \,\approx\, \frac{{{\int}_0^{r_0} {T\left( r \right)q\left( r \right)rdr} }}{{{\int}_0^{r_0} {q\left( r \right)rdr} }}.$$

### DFT calculations

To determine the energy barrier between ABA and ABC stacking structures, we performed a CI-NEB calculation based on DFT with two different types of exchange-correlation functionals: local density approximation (LDA) and meta-GGA (SCAN) + rVV10 in the Quantum-ESPRESSO package^54–57^. The SCAN + rVV10 functional considers the nonlocal interactions, including van der Waal forces between difference layers and many-body effects in electrons, resulting in more accurate energies and structures compared with the traditional GGA functional. A norm-conserved pseudopotential was implemented in the calculation, and the kinetic energy cutoff was set as 50 Ry, while the density cutoff was set as 200 Ry^58^. The Brillouin zone of a 2 × 2 supercell with 20 Å vacuum separation was sampled using a 6 × 6 × 1 Monkhorst–Pack grid with Methfessel–Paxton smearing of 0.01 Ry and the 2D cutoff^59–61^. A 12 × 12 × 1 Monkhorst–Pack grid case calculation was conducted, yielding the same results as 6 × 6 × 1. The optimized atomic positions with the maximum force on any atom <0.001 a.u. was implemented with an initial interlayer separation at the experimental value of 3.36 Å along the *z-*axis for TLG. To determine the energy barrier between ABA and ABC structures, we also performed a CI-NEB calculation. A 2 × 2 supercell (including 24 carbon atoms) was calculated with both LDA and SCAN + rVV10 exchange-correlation models in the CI-NEB process. It should be mentioned that the LDA functional will overestimate the interactions regarding the van der Waals dispersion, but the long-range correlation will be lost, which is included in the SCAN functional^62^.

## Supplementary information

supplementary information
