# Supplementary material for: Light-induced irreversible structural phase transition in trilayer graphene
Source: Light Sci Appl. 2020 Oct 13;9:174. doi: 10.1038/s41377-020-00412-6 (PMC7553909; doi:10.1038/s41377-020-00412-6)

**Supplementary Information for**

**Light-induced irreversible structural phase transition in trilayer graphene**

Jianyu Zhang^1,#^, Jinsen Han^1,#^, Gang Peng^1,#^, Xi Yang^2^, Xiaoming Yuan^3^, Yongjun Li^4^, Jianing Chen^5,6,7^, Wei Xu^2^, Ken Liu^2^, Zhihong Zhu^2^, Weiqi Cao^8^, Zheng Han^9,10,11^, Jiayu Dai^1,*^, Mengjian Zhu^2,*^, Shiqiao Qin^2^_,_ Kostya S. Novoselov^8,12^

^1^ Department of Physics, National University of Defense Technology, Changsha 410073, China

^2^ College of Advanced Interdisciplinary Studies, National University of Defense Technology, Changsha 410073, China

^3^ Hunan Key Laboratory of Super Micro-structure and Ultrafast Process, School of Physics and Electronics, Central South University, Changsha 410083, China

^4^ Quantum Design China (Beijing) Co., Ltd, Beijing 100015, China

^5^ Beijing National Laboratory for Condensed Matter Physics, Institute of Physics, Chinese Academy of Sciences, Beijing 100190, China

^6^ School of Physical Sciences, University of Chinese Academy of Sciences, Beijing 100049, China

^7^ Songshan Lake Materials Laboratory, Dongguan, Guangdong 523808, China

^8^ Chongqing 2D Materials Institute, Liangjiang New Area, Chongqing 400714, China

^9^ Shenyang National Laboratory for Materials Science, Institute of Metal Research, Chinese Academy of Sciences, Shenyang 110016, China

^10^ School of Material Science and Engineering, University of Science and Technology of China, Anhui 230026, China

^11^ State Key Laboratory of Quantum Optics and Quantum Optics Devices, Institute of Opto-Electronics, Shanxi University, Taiyuan 030006, China

^12^ Department of Materials Science and Engineering, National University of Singapore, Singapore 117575, Singapore

^#^ These authors contributed equally to this work

^*^ To whom correspondence should be addressed. Email: [jydai@nudt.edu.cn](mailto:jydai@nudt.edu.cn); [zhumengjian11@nudt.edu.cn](mailto:zhumengjian11@nudt.edu.cn)

**Figure S1.** Raman maps of the full width at half maximum (FWHM) of TLG.

**Figure S2.** Raman maps of the integrated G band intensity of additional TLG samples.

**Figure S3.** Raman maps of the integrated 2D band intensities of TLG.

**Figure S4.** Light-induced structural phase transition in pure ABC-stacked regions.

**Figure S5.** Light-induced structural phase transition in an additional TLG sample.

**Figure S6.** s-SNOM imaging of the domain walls in multilayer graphene before and after additional laser irradiation.

**Figure S7.** Evolution of the G band and 2D band Raman shift of TLG on Si/SiO_2_ substrate under laser irradiation.

**Figure S8.** Light-induced structural phase transition in TLG on Al_2_O_3_ substrate.

**Figure S9.** Light-induced structural phase transition in TLG with different laser exposure time.

**Figure S10.** Light-induced structural phase transition in TLG by 785 nm laser irradiation.

**Figure S11.** Spatially resolved temperature distribution in TLG under laser irradiation.

**Figure S12.** Laser-induced defects at the edges of TLG.

**Table S1.** Comparison of the calculated total energies of TLG for ABA, ABC and ABX stacking structures by DFT calculations.


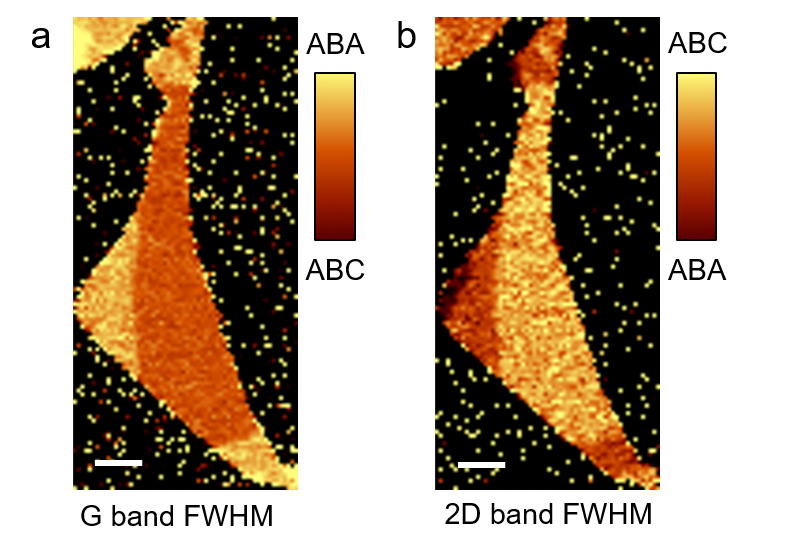


**Figure S1.** Raman maps of the full width at half maximum (FWHM) of TLG. **a,** G band FWHM. **b,** 2D band FWHM of sample #2. The scale bars are 4 μm.


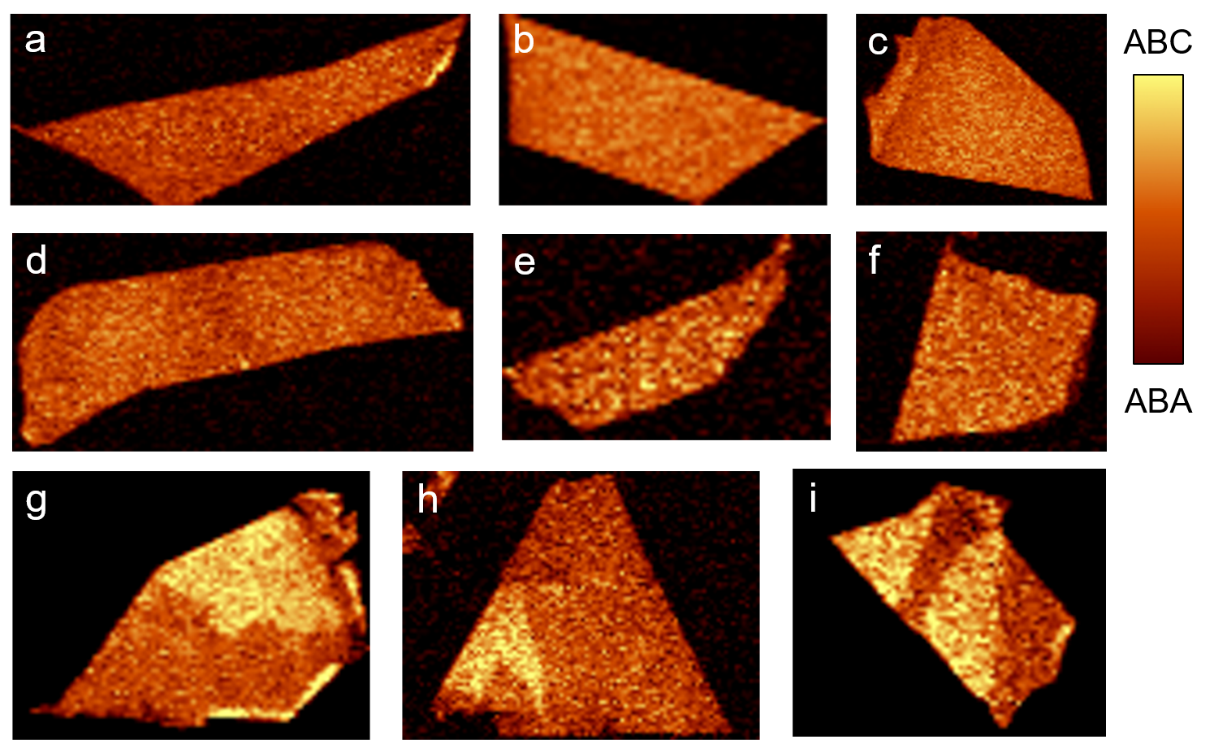


**Figure S2.** Raman maps of the integrated G band intensity of additional TLG samples. **a-f,** typical pure ABA-stacked TLG. **g-i,** Additional TLG samples containing both ABA-stacked domains (darker regions) and ABC-stacked domains (brighter regions).


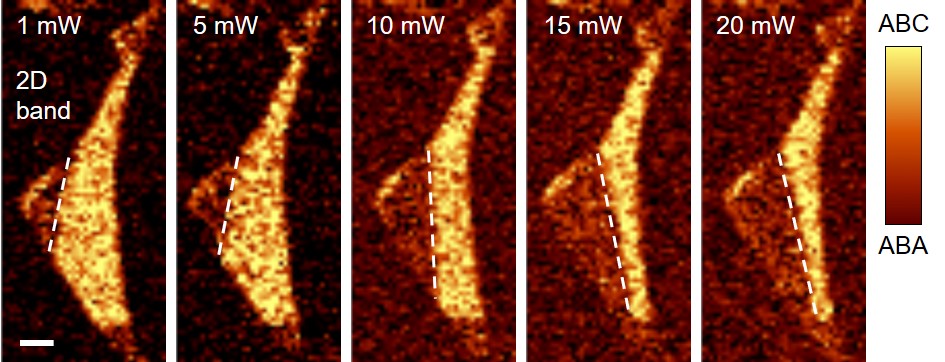


**Figure S3.** Raman maps of the integrated 2D band intensities of TLG. Measured Raman mapping of integrated 2D band intensities from sample #2 after laser irradiation at various laser power from 1 mW to 20 mW. The white dashed lines indicate the gradual movement of the ABA/ABC domain wall under laser irradiation. The scan direction of laser beam is from left to right and from top to bottom. The scale bar is 4 μm.


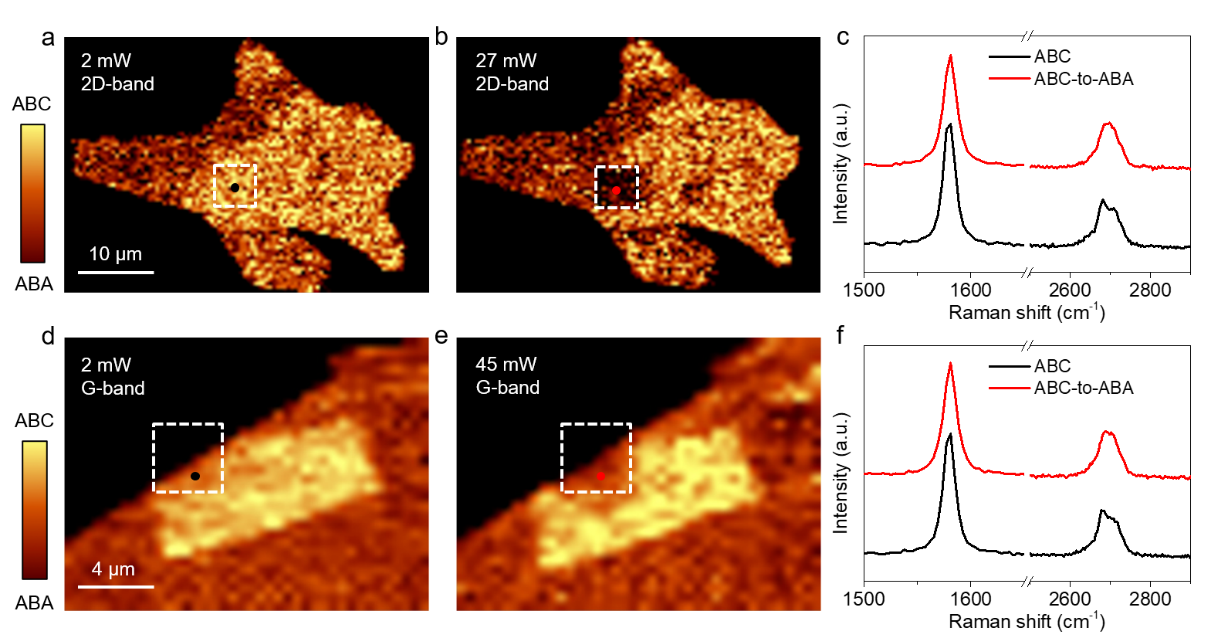


**Figure S4.** Light-induced structural phase transition in pure ABC-stacked regions. Raman maps of 2D band of a TLG sample #117 before laser irradiation (a) and after laser irradiation (b). The laser power is 27 mW and the exposure time 30 min. (c) Raman spectrum of TLG before and after laser irradiation. The data are taken from the black and red solid dots marked in (a) and (b). Raman maps of G band of a TLG sample #116 before laser irradiation (d) and after laser irradiation (e). The laser power is 45 mW and the exposure time 25 min. (f) Raman spectrum of TLG before and after laser irradiation. The data are taken from the black and red solid dots marked in (d) and (e).


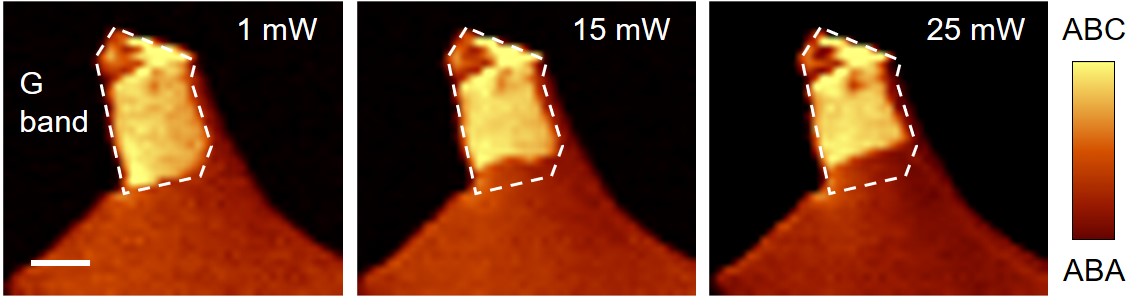


**Figure S5.** Light-induced structural phase transition in an additional TLG sample. Raman mappings of integrated G band intensity of TLG from sample #65 after laser irradiation at various laser power from 1 mW to 20 mW. The white dashed shapes indicate the initial geometry of ABC-stacked domain before laser irradiation. It is clear that the structural phase transition starts from the domain walls under laser irradiation. The scan direction of laser beam is from left to right and from top to bottom. The scale bar is 10 μm.


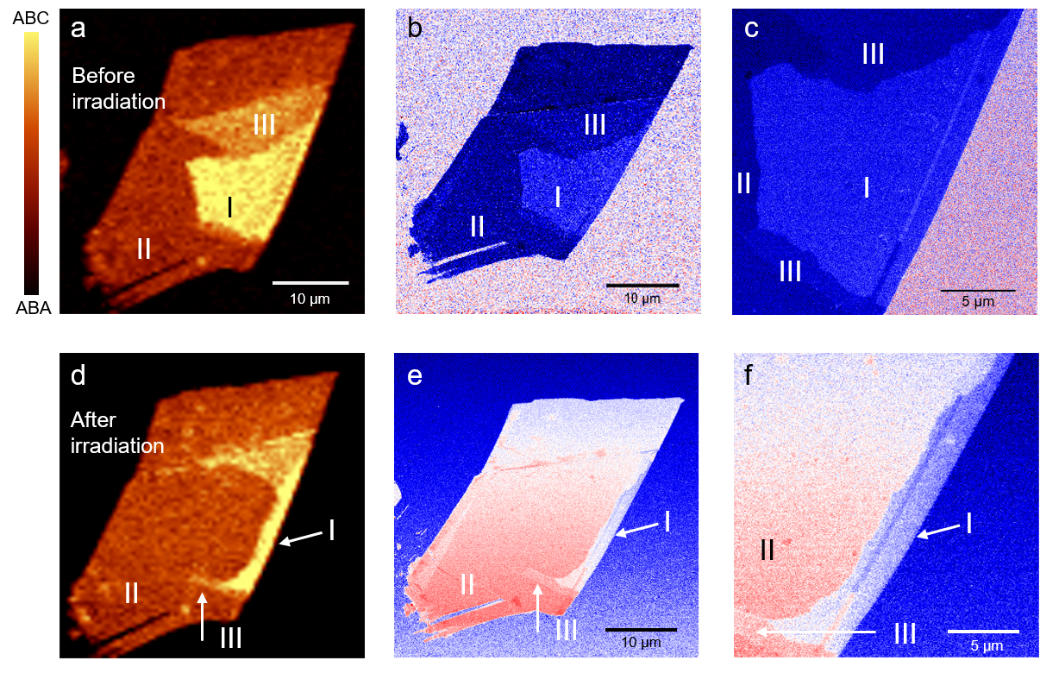


**Figure S6.** s-SNOM imaging of the domain walls in multilayer graphene before and after additional laser irradiation. (a) Raman maps of integrated G band of graphene before and (d) after laser irradiation. The laser power is 20 mW and the exposure time 17 min. Region I, II and III correspond to ABC, ABA and mixed ABC+ABA stacking in few-layer graphene, respectively. (b) The SNOM images of graphene before laser irradiation and (e) after laser irradiation. (c) and (f) The zoomed-in SNOM images before and after laser irradiation.


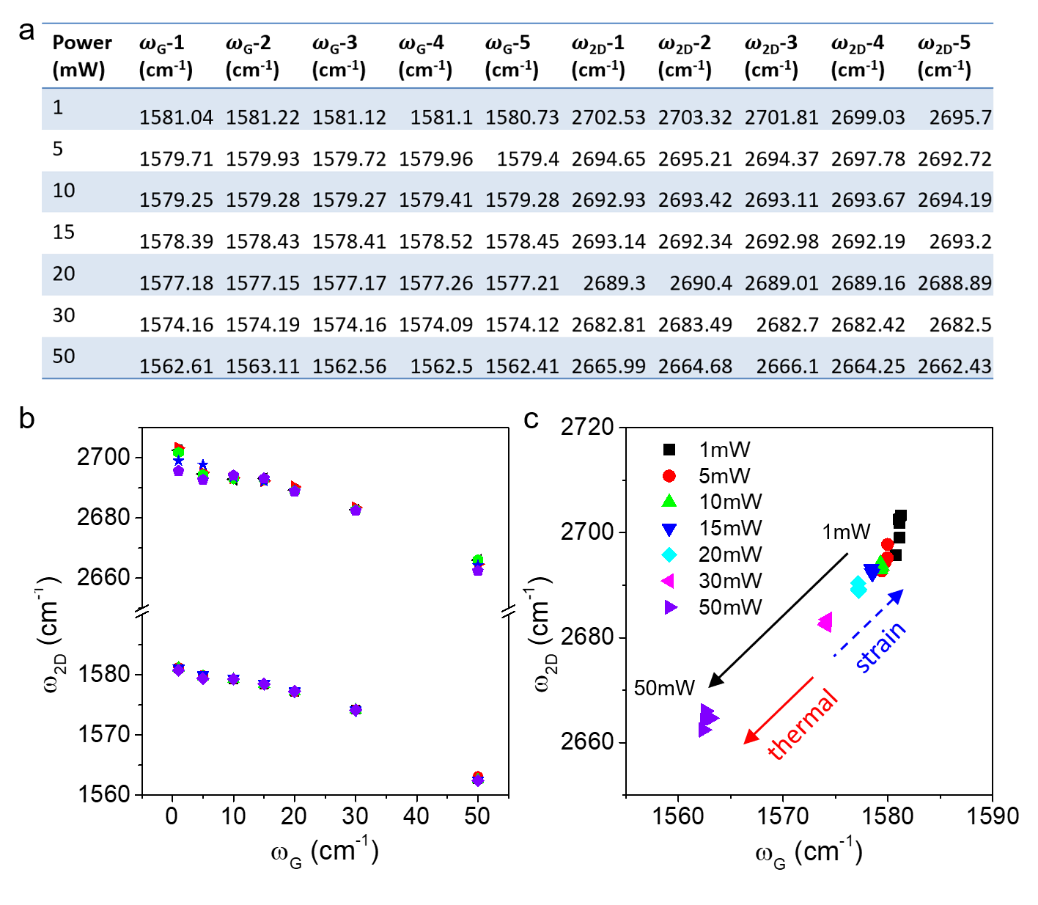


**Figure S7.** Evolution of the G band and 2D band Raman shift of TLG on Si/SiO_2_ substrate under laser irradiation. (a) $\omega$_G_ and $\omega$_2D_ of TLG under laser irradiation with power from 1 mW to 50 mW. Data are taken from 5 different pixels in the Raman map in Figure 2 maintext. (b) $\omega$_G_ and $\omega$_2D_ as a function of laser power. (c) $\omega$_G_ *vs.* $\omega$_2D._ The black arrow shows the downshift in both$\omega$_G_ and $\omega$_2D_ of TLG under laser irradiation, while the red and blue arrows indicate the thermal-induce downshift and strain-induced upshift, respectively.


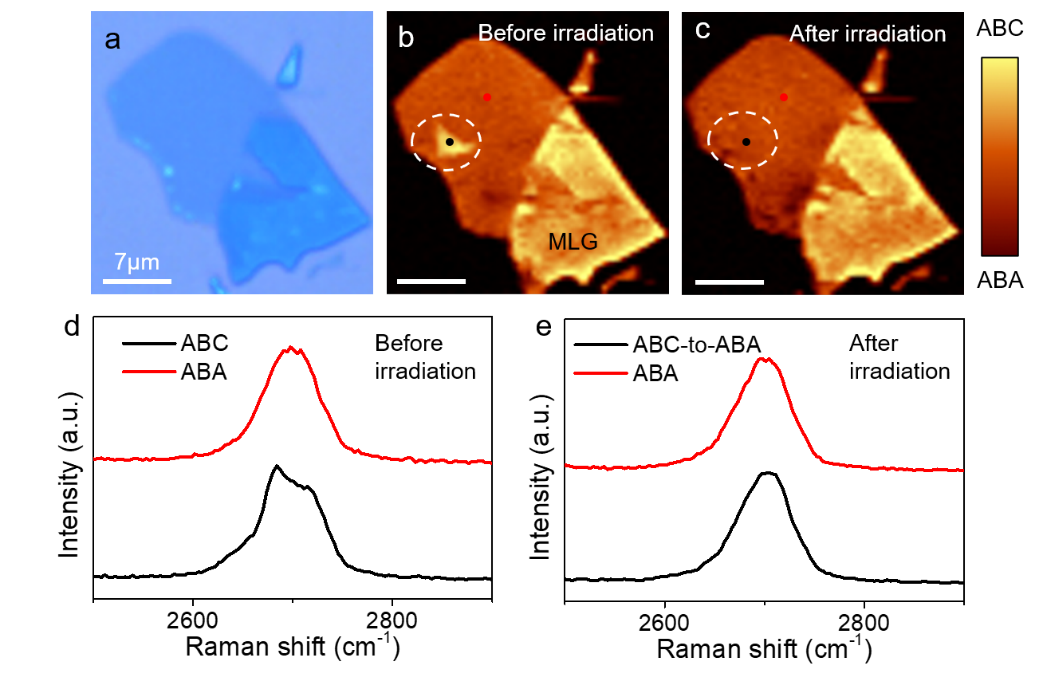


**Figure S8.** Light-induced structural phase transition in TLG on Al_2_O_3_ substrate. (a) Optical microscopy image of a graphene sample #211. Raman maps of integrated G peak of graphene on Al_2_O_3_/SiO_2_/Si substrate before laser irradiation (b) and after laser irradiation (c). The laser power is 40 mW and exposure time is 10 min. The scale bar is 7 μm. The white dashed zones in (b) highlight the ABC-stacked TLG domain before laser irradiation, while it completely transforms to ABA stacking after laser irradiation in (c). Raman spectrum of different graphene regions taken from the marked solid dots in TLG before (d) and after (e) laser irradiation.


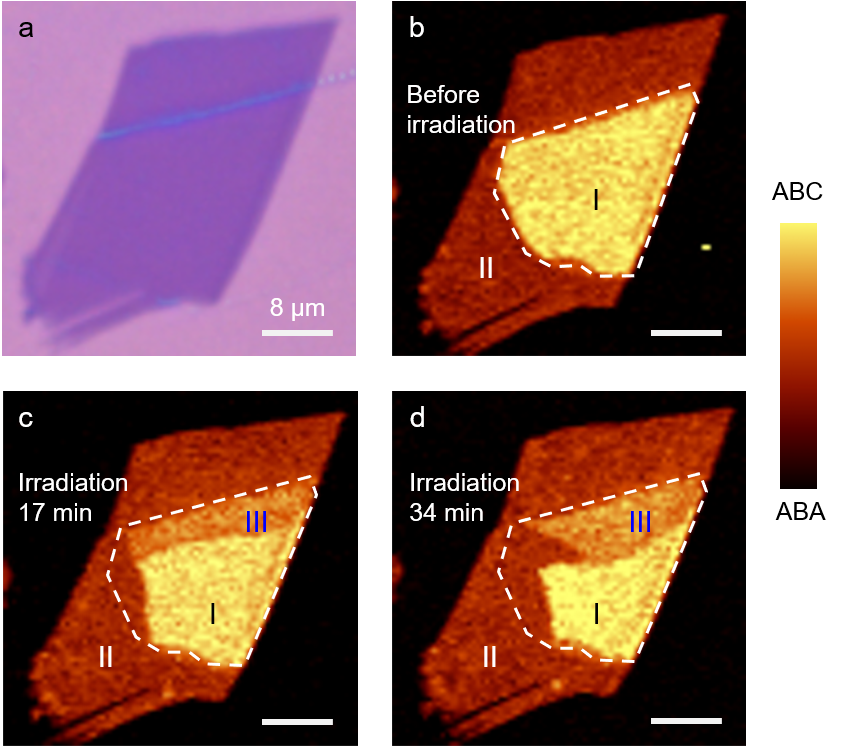


**Figure S9.** Light-induced structural phase transition in TLG with different laser exposure time. (a) optical microscopy image. Raman maps of integrated G peak intensity before laser irradiation (b) and after laser irradiation with different exposure time 17min (c) and 34 min (d), respectively. The white dashed polylines highlight the original region of ABC domain. Region I, II and III correspond to ABC stacking, ABA stacking and mixed ABA and ABC stacking domains, respectively.


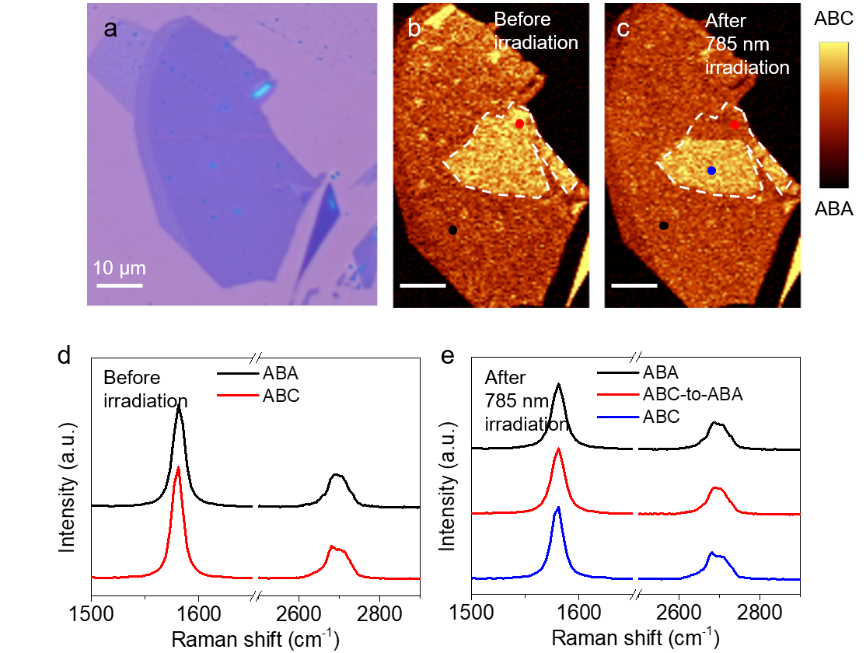


**Figure S10.** Light-induced structural phase transition in TLG by 785 nm laser irradiation. (a) optical microscopy image. Raman maps of integrated G peak intensity before laser irradiation (b) and after laser irradiation (c). The wavelength of applied laser is 785 nm, the laser power and spot size of laser beam is 40 mW and ~ 10 μm^2^, and the exposure time is 60 min, respectively. The white dashed polylines highlight the original region of ABC domain. (d) Raman spectrum of different graphene regions taken from the marked dots before laser irradiation (d) and after laser irradiation (e).


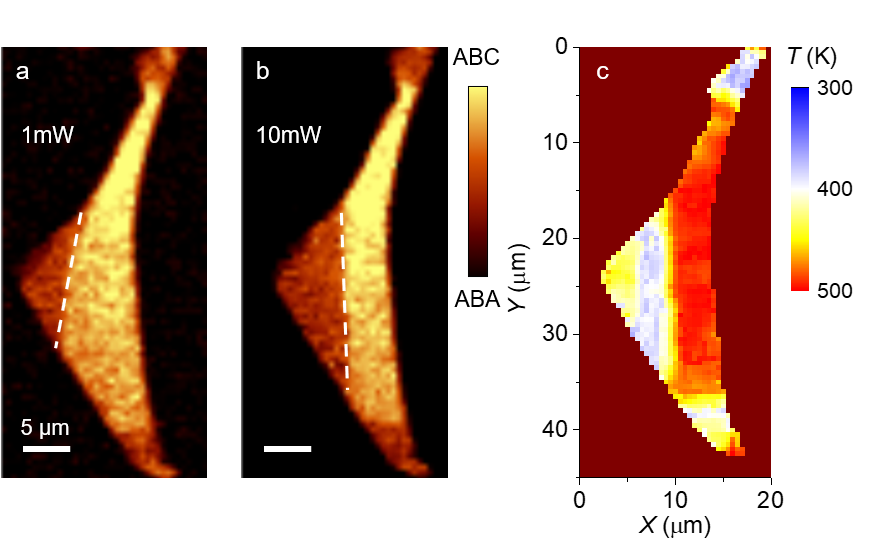


**Figure S11.** Spatially resolved temperature distribution in TLG under laser irradiation. (a) Raman map of G peak before laser irradiation and (b) after laser irradiation. The laser power is 10 mW and the exposure time 12 min. The white dashed lines indicate the changing domain wall between ABC and ABA regions. (c) The spatial distribution of temperature in graphene determined from the Raman maps.


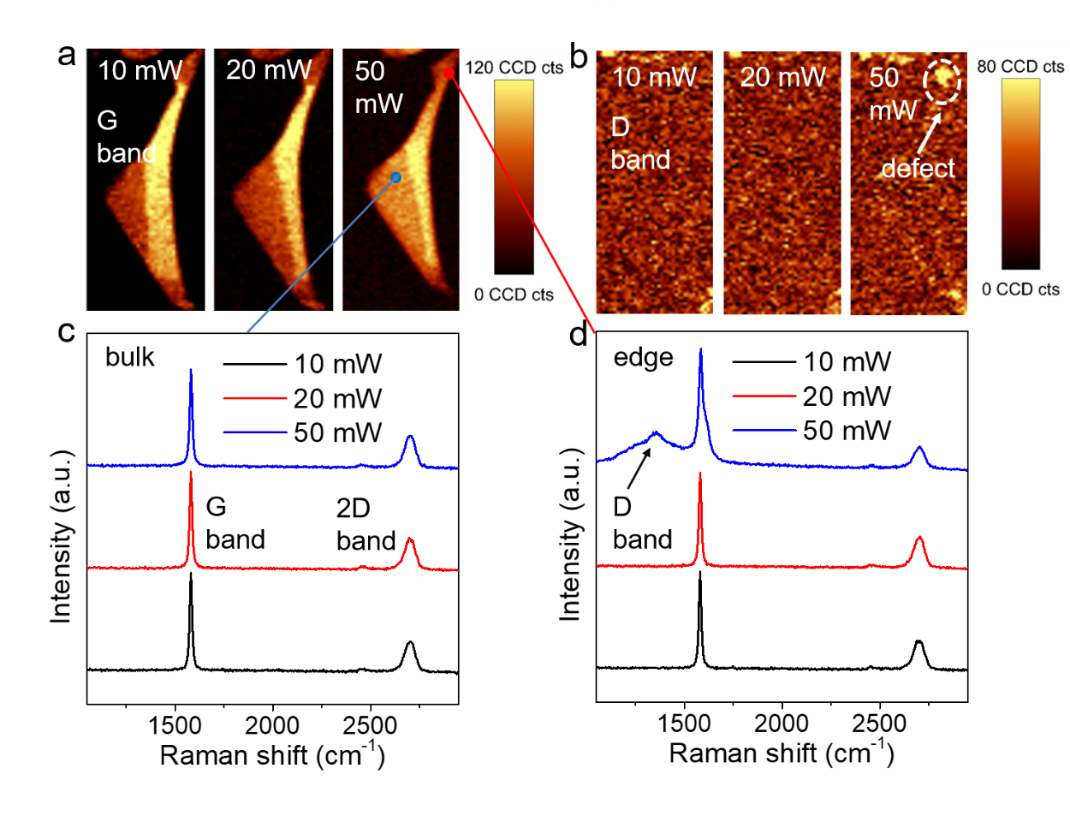


**Figure S12.** Laser-induced defects at the edges of TLG. **a,** Raman mappings of G band intensity of sample #2 under laser irradiation with power from 10 mW to 50 mW. **b,** Raman mappings of D band intensity showing laser-induced defects at the edge of sample at 50 mW laser illumination, as marked by white dashed zones. **c,d,** Raman spectra of TLG taken from the bulk and edge in the sample. The D band appears at the edge under 50 mW laser illumination, highlighted by the arrow in (**d**).

**Table S1.** Comparison of the calculated total energies (eV) of TLG for ABA, ABC and ABX stacking structures by DFT calculations with SCAN+rVV10 functional and LDA functional, respectively.


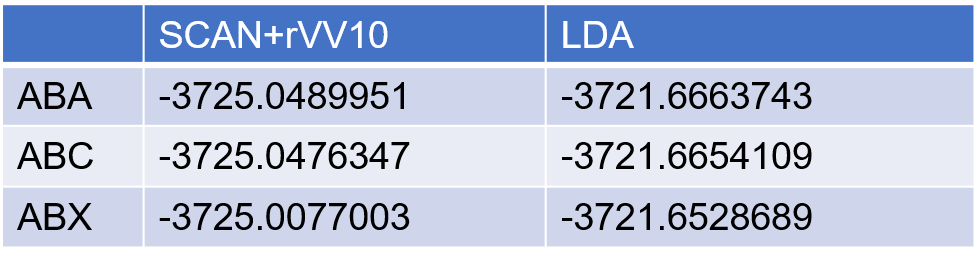

Supplement: Supplementary file 1 — supplementary information [file 41377_2020_412_MOESM1_ESM.docx]
